# Penetrating potential evaluation of cyclotriphosphazenes target blood-brain barrier via computational toxicology methods

**DOI:** 10.1016/j.isci.2026.117147

**Published:** 2026-08-13

**Authors:** Qiyu Yang, Jinming Zhang, Yuxuan Dao, Zhengrui He, Rui Lu, Rummana Jaman, Yaole Wu, Shuqi Liu, Conglin Zhang, Zhibi Zhang, Jiaqi Zhou

**Affiliations:** 1 School of Basic Medical Sciences, Dali University, Dali, Yunnan 671000, China; 2 College of Clinical Medicine, Dali University, Dali, Yunnan 671000, China; 3 College of Sports Science, Dali University, Dali, Yunnan 671000, China; 4 Yunnan Key Laboratory of Breast Cancer Precision Medicine, Academy of Biomedical Engineering, Kunming Medical University, Kunming, Yunnan 650500, China

**Keywords:** cyclotriphosphazenes, toxicological evaluation, blood-brain barrier, computational toxicology methods

## Abstract

Cyclotriphosphazenes (CTPs), widely used as lithium-ion battery electrolyte additives, are increasingly recognized as environmental contaminants with potential neurotoxicity. We developed an integrated computational framework to evaluate the blood-brain barrier (BBB) penetration potential and neurotoxic mechanisms of CTPs. Six representative CTPs were predicted to penetrate the BBB via passive diffusion and impair BBB function. Among them, HPCTP, PFPCTP, HCCTP, and HFCTP primarily disrupted BBB function through inhibition of ATP-binding cassette transporters. HPCTP exhibited the strongest BBB-disrupting potential, primarily through interactions with SLC2A1 and SLC6A3 and through the modulation of BBB-related gene expression. Experimental validation in hCMEC/D3 cells showed that HPCTP impaired glucose transport and utilization and significantly downregulated SLC2A1 expression. These findings provide a strategy for assessing BBB penetration of exogenous chemicals and highlight the potential CNS risks associated with environmental CTP exposure.

## Highlights

• Six cyclotriphosphazenes were evaluated for BBB penetration potential
• Computational analysis revealed HPCTP as the strongest BBB-disrupting compound
• HPCTP interacted with SLC2A1 and SLC6A3 transporters at the BBB
• HPCTP impaired glucose transport and reduced SLC2A1 expression in hCMEC/D3 cells

## Graphical abstract

**Figure:**
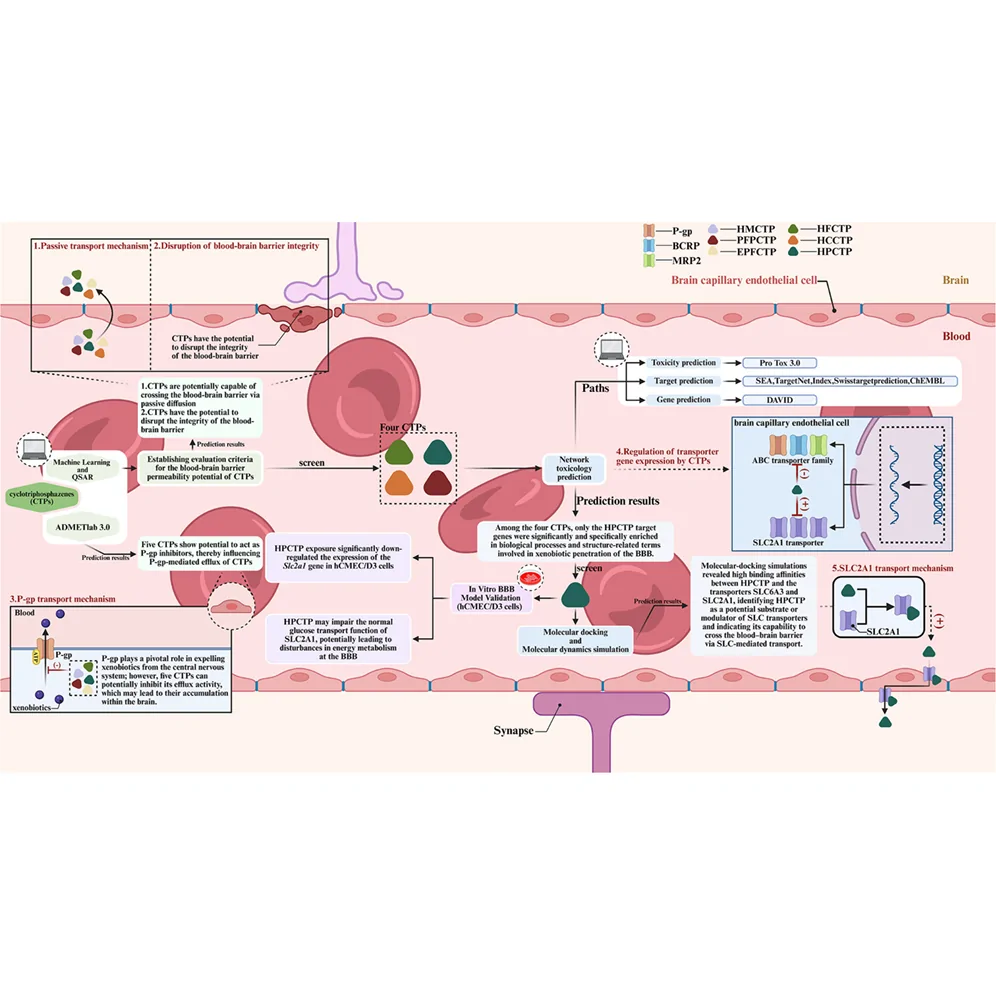

## Teaser

Computational toxicology; Environmental toxicology

## Introduction

With the rapid advancement of industries such as new energy vehicles and consumer electronics, the demand for high-performance lithium-ion batteries in industrial applications has increased significantly. Consequently, extensive research efforts have been directed toward improving lithium battery performance through the use of electrolyte additives.^1^^,^^2^ Among these, cyclotriphosphazenes (CTPs) represent one of the most important classes of such electrolyte additives. Their key advantages include highly efficient overcharge protection, excellent electrochemical compatibility, superior thermal stability, and significant flame retardancy. Owing to these beneficial properties, CTPs significantly enhance the safety, longevity, and overall performance of lithium batteries, leading to their widespread inclusion in various battery products. Moreover, hexaphenoxycyclotriphosphazene (HPCTP) is considered a promising alternative to the conventional organophosphorus flame retardant triphenyl phosphate.^3^^,^^4^^,^^5^^,^^6^^,^^7^

**Figure 1:**
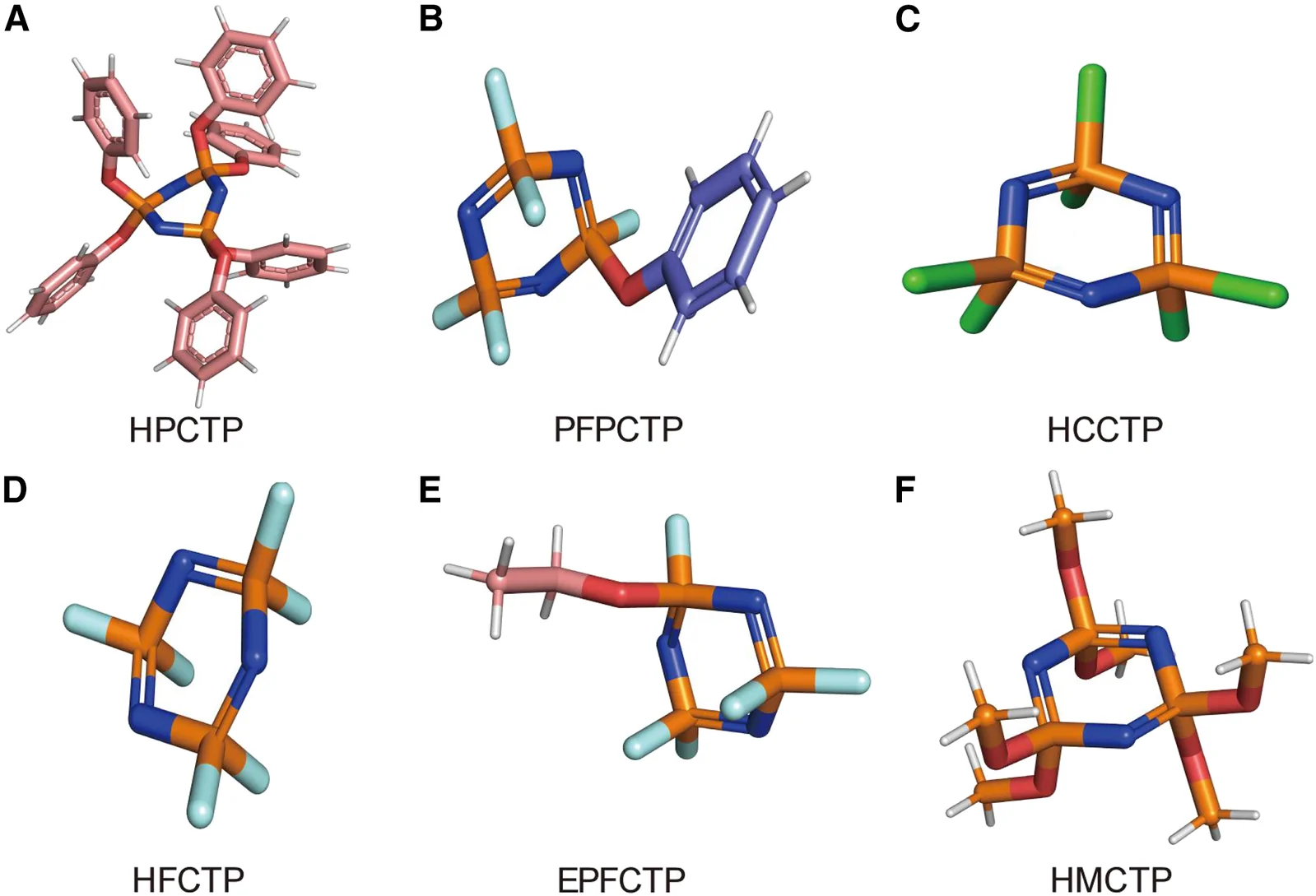
Three-dimensional structures of the six CTPs (A) HPCTP. (B) PFPCTP. (C) HCCTP. (D) HFCTP. (E) EPFCTP. (F) HMCTP.

In industrial production, CTPs are functionalized by substituting different groups onto the phosphorus atoms within the hexacyclic structure, which is composed of alternating nitrogen and phosphorus atoms, to meet diverse performance requirements as electrolyte additives.^8^ Commercially prevalent CTP derivatives include fluorinated hexafluorocyclotriphosphazene (HFCTP), chlorinated hexachlorocyclotriphosphazene (HCCTP), phenoxy-substituted HPCTP, alkoxy-substituted hexamethoxycyclotriphosphazene (HMCTP), as well as mixed-substituent derivatives such as pentafluoro(phenoxy) cyclotriphosphazene (PFPCTP) and ethoxy(pentafluoro) cyclotriphosphazene (EPFCTP) (Figure 1). Among these, HCCTP serves as a key precursor for the synthesis of various cyclotriphosphazene derivatives.^3^^,^^7^^,^^9^^,^^10^^,^^11^^,^^12^^,^^13^^,^^14^^,^^15^

Recent environmental investigations have demonstrated the widespread occurrence of CTPs in multiple environmental media. Six common CTPs have been detected in both surface water and sediment samples, with total concentrations (ΣCTPs) in surface water ranging from 4.1 to 556 ng/L and a mean value of 124 ng/L. Among individual compounds, HCCTP and EPFCTP exhibited relatively high detection frequencies of up to 90% and 81%, respectively, whereas other CTPs were detected at lower but still measurable levels. In sediments, detection frequencies of individual CTPs ranged from 19% to 95%. EPFCTP and HPCTP were the dominant compounds, with mean concentrations of 24 and 20 ng/g dry weight (dw).^16^

In addition to aquatic environments, a recent urban environmental study reported the presence of all six CTPs in dust samples collected from various locations in Guangzhou, China, including homes (11.9–122 ng/g; median 50.5 ng/g), air conditioner filters (75.7–321 ng/g; median 142 ng/g), e-waste recycling plants (24.0–1790 ng/g; median 150 ng/g), major roads (1.95–56.3 ng/g; median 4.94 ng/g), and underground parking garages (3.38–142 ng/g; median 25.0 ng/g). Notably, exposure concentrations at e-waste recycling plants were substantially higher than those observed in other urban settings. Among the analyzed compounds, HPCTP was identified as the most abundant compound, consistent with its large-scale production and widespread application.^17^

Although detailed production statistics, such as the global output of CTPs used in the lithium-ion battery industry, remain limited,^18^ these findings collectively provide initial evidence of the widespread occurrence and persistent environmental accumulation of CTPs, suggesting that their production and consumption are likely to continue increasing in response to the growing demand for lithium-ion batteries.

Concerning human exposure, only one study conducted in Hangzhou, China, has reported detection of CTPs in human urine. HFCTP (mean 1.2 ng/mL, range:< LOD −7.7 ng/mL) and EPFCTP (mean 0.96 ng/mL, range:< LOD −11 ng/mL) were identified as the predominant CTPs, with detection frequencies of 79%–84% for HFCTP, EPFCTP, and HMCTP. Currently, reference materials and exposure risk data for CTPs remain highly limited.^19^ In terms of toxicity and mechanisms, one study demonstrated that HPCTP induced eye malformations, body deformities, and early embryonic mortality in Japanese Medaka by antagonizing retinoic acid X receptors and retinoic acid receptors.^18^ Additionally, HPCTP caused developmental defects in *Danio rerio*, including reduced body length and cardiac malformations, and induced dose-dependent depressive-like behavior via suppression of serotonin 1A (5-HT_1_A) receptor signaling.^20^ Furthermore, long-term combined exposure to six CTPs (PFPCTP, EPFCTP, HFCTP, HPCTP, HMCTP, and HCCTP) at environmentally relevant concentrations resulted in pathological lung injuries in mice, such as alveolar destruction and pulmonary fibrosis, as well as mitochondrial dysfunction in BEAS-2B cells.^21^

Although representative CTPs, including PFPCTP, EPFCTP, HFCTP, HPCTP, HMCTP, and HCCTP, are listed in the PubChem database, most remain unregistered as chemicals in China, Japan, the EU, and the US.^17^ Moreover, while HPCTP is considered an alternative to triphenyl phosphate, its toxicological safety requires comprehensive evaluation. These factors collectively underscore the considerable challenges in environmental regulation of this emerging class of substances.

To summarize, there is a pressing need to establish a comprehensive and systematic evaluation framework to assess the entire life cycle of cyclotriphosphazenes (CTPs). This framework should encompass their environmental behavior, occurrence, biological exposure pathways, bioaccumulation potential, toxic effects, and underlying mechanisms. Such an approach will facilitate a balanced consideration of their industrial benefits against potential environmental risks. Thus, we have employed an integrated methodology combining machine learning, quantitative structure-activity relationship (QSAR) modeling, computational prediction, and network toxicology approaches to perform a preliminary investigation to evaluating the potential of HPCTP, PFPCTP, HCCTP, HFCTP, EPFCTP, and HMCTP to penetrate into the blood-brain barrier (BBB) and central nervous system (CNS) toxicity. The findings are expected to advance our understanding of the toxicological profiles and mechanisms of these compounds, thereby providing a foundation for subsequent in-depth research.

## Results

### Predicted potential effects of CTPs on the BBB and passive diffusion penetration properties

#### The potential BBB impairment of CTPs

**Figure 2:**
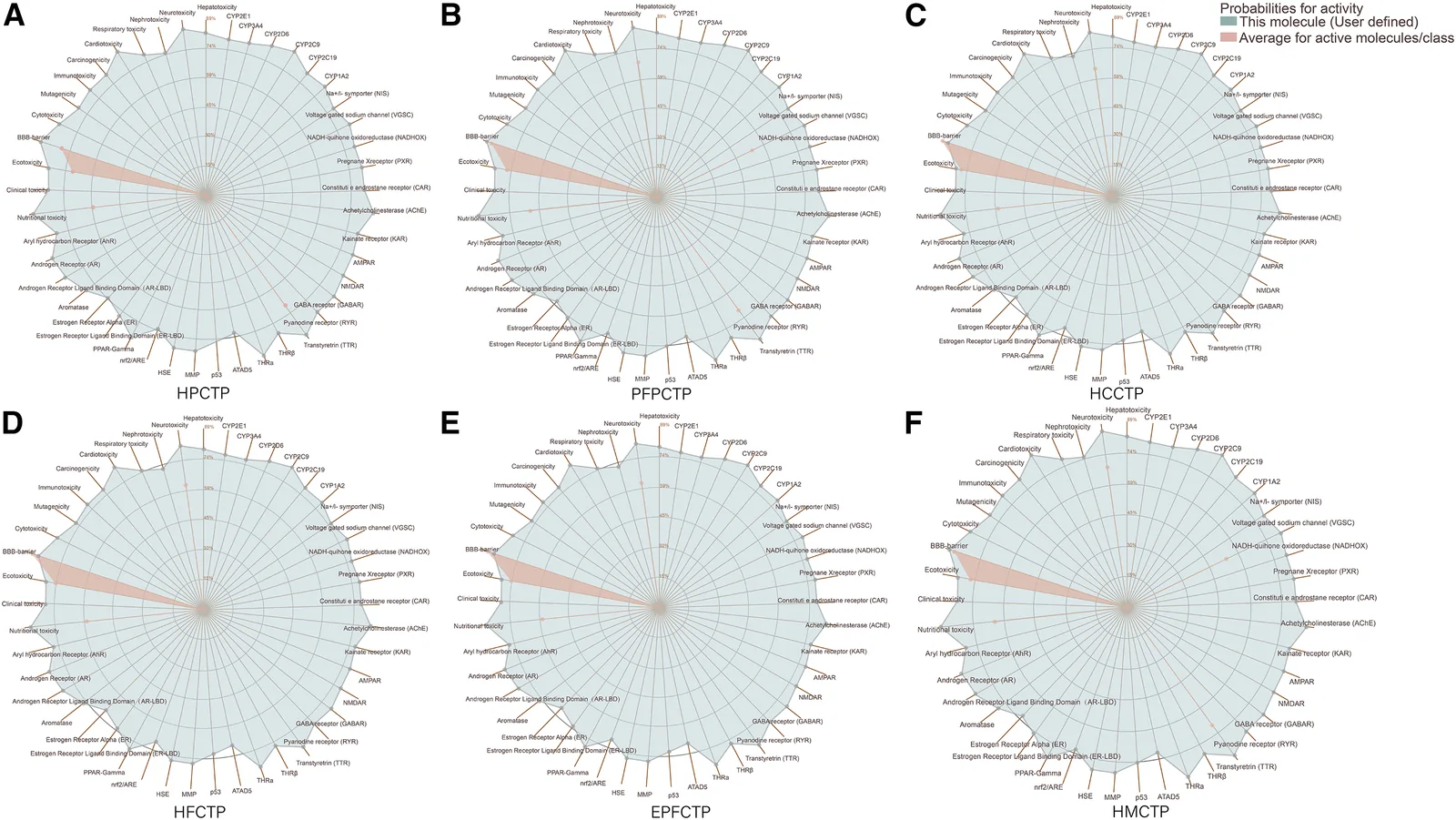
Radar plots of predicted toxicity profiles for six CTPs (A**)** HPCTP, (B) PFPCTP, (C) HCCTP, (D) HFCTP, (E) EPFCTP, and (F) HMCTP. Each radar plot illustrates the probability scores across multiple pharmacological activity classes, where the red sector represents the molecule-specific prediction and the green region denotes the activity range for known active compounds.

The structural formulas of the six CTPs were submitted to the ProTox 3.0 toxicity-prediction platform with all available predictive models activated, and data were visualized to generate the corresponding radar plots (Figure 2). The predictive outcomes suggest that all six CTPs possess the potential to interact with multiple receptors concurrently, and display particularly notable effects on the BBB. Within the ProTox 3.0 framework, receptors such as matrix metalloproteinase (MMP), peroxisome proliferator-activated receptor γ (PPAR-γ), the aryl hydrocarbon receptor (AhR), estrogen receptor alpha (ERα), estrogen receptor ligand-binding domain (ER-LBD), androgen receptor (AR), pregnane X receptor (PXR), constitutive androstane receptor (CAR), and CYP3A4 are currently recognized as key predictors of the potential of xenobiotics to penetrate the BBB. Moreover, all six CTPs exhibit toxicity probabilities exceeding 74% for eight of these receptors, excluding ERα.

These findings indicate that CTPs are highly likely to achieve BBB penetration by modulating these receptors and by impairing BBB integrity.

#### CTPs potentially penetrate the BBB via passive diffusion

As described in predicted potential effects of CTPs on the BBB and passive diffusion penetration properties, the ability of compounds to cross the BBB via passive diffusion may vary depending on their physicochemical properties, and toxicity prediction tools can effectively predict these characteristics.^22^^,^^23^^,^^24^ Following computational prediction of the six CTPs using the Maestro software package, 12 evaluation parameters were compiled and scored, according to the criteria established in Table 1. As summarized in Table 2, all six CTPs achieved scores of 8 or higher according to the scoring system described in predicted potential effects of CTPs on the BBB and passive diffusion penetration properties, suggesting their potential for passive diffusion across the BBB. It is worth noting that QPlogBB is regarded as the parameter most strongly associated with BBB penetration capability among these molecular descriptors. In this study, five CTPs (excluding HCCTP) exhibited QPlogBB values within the favorable range for BBB permeability (−3 to 1.2). Additionally, the calculated molecular weights of five compounds (excluding HPCTP) remained below the recommended threshold of 450 g/mol for CNS-targeting drugs (Table 2). Furthermore, these compounds displayed fewer than 5 hydrogen bond (HB) acceptors (acceptor HB) and completely lacked hydrogen bond donors (donor HB). This molecular characteristic favors desolvation prior to interaction with the lipophilic regions of cell membranes, thereby facilitating passive diffusion.^27^^,^^29^

**Table 1:** Descriptors of BBB-related molecular scores and their reference ranges

Molecular descriptors	Range for BBB	Comments	Reference
QPlogBB	−3–1.2	predicted brain/blood partition coefficient. predicts favorable BBB passive permeation for molecules, which range between −3.0 and 1.2 (based upon 95% of known drugs)	Eigenmann et al.^25^; Kelder et al.^26^
QPPMDCK	>500 nm/s	predicted MDCK cell line permeability in nm/s; MDCK cells are considered to be a good mimic for the BBB; above the recommended is consider great based upon 95% of known drug	Eigenmann et al.^25^
MW	<450 g/mol	molecular weight; compounds with high molecular weight are unable to passively cross the blood brain barrier; this can be related with volume	van de Waterbeemd et al.^27^
QPlogPo/w	4	predicted octanol/water partition coefficient	Kelder et al.^26^
Dipole	1–12.5	computed dipole moment of the molecule	Figueira et al.^28^
Volume	500–2,000 A^3^	total solvent-accessible volume in cubic angstroms	Figueira et al.^28^
Donor HB	0	estimated number of hydrogen bonds that would be donated by the solute to water molecules in an aqueous solution; no hydrogen bond donors facilitates their desolvation before entering the lipophilic phase of the cell membranes	Eigenmann et al.^25^
Acceptor HB	<5	estimated number of hydrogen bonds that would be accepted by the solute from water molecules in an aqueous solution; low effective number of H-bond acceptors (<5) facilitates their desolvation before entering the lipophilic phase of the cell membranes	Eigenmann et al.^25^
QPPcaco	>500 nm/s	predicted apparent Caco-cell permeability in nm/s; above the recommended is consider great based upon 95% of known drugs	Eigenmann et al.^25^
QPlogKhsa	−1.5 to 1.5	prediction of binding to human serum albumin	Figueira et al.^28^
PSA	70–90	van der Waals surface area of polar nitrogen and oxygen atoms and carbonyl carbon atoms; recommended below the thresholds of 90 Å2 (ref. a) and 70 Å2 (ref-b)	van de Waterbeemd et al.^27^; Kelder et al.^26^
Rotatable bonds	<6	number of non-trivial (not CX3), non-hindered (not alkene, amide, small ring) rotatable bonds; less than 6 bonds recommended for CNS drugs	Brito-Sánchez et al.^29^

**Table 2:** Molecular descriptors and BBB scores predicted by Schrödinger software

Molecule	QPlogBB	QPPMDCK	MW	QPlogPo/w	Dipole	Volume	DonorHB	AccptHB	QPPCaco	QPlogKhsa	PSA	#rotor	Score
HPCTP	−0.5	1,602.261	693.571	5.123	8.279	1,880.919	0	14.5	2,825.122	−0.14	72.975	8	8
PFPCTP	0.584	10,000	323.038	2.915	1.945	773.453	0	4.5	3,487.27	−0.425	72.509	2	11
HCCTP	1.279	10,000	347.659	3.149	0.004	722.046	0	4.5	4,745.452	−0.49	58.279	0	8
HFCTP	0.812	10,000	248.932	1.32	0.005	534.87	0	4.5	2,789.85	−1.033	68.285	0	9
EPFCTP	0.605	10,000	274.994	2	2.436	664.134	0	4.5	3,440.229	−0.743	73.19	2	11
HMCTP	0.102	4,446.693	321.146	2.921	0.042	904.371	0	4.5	7,626.467	−0.215	86.485	6	9

To enhance prediction accuracy regarding the BBB penetration potential of CTPs, we integrated computational results from both Schrödinger software and the ADMETlab 3.0 database. A total of 10 molecular descriptors associated with BBB permeability were calculated for the six CTPs (Table 3). Among these descriptors, particular attention was given to the parameter labeled “BBB.” According to the official documentation, this metric quantifies the predicted probability (range: 0–1) of a chemical substance crossing the BBB.^30^ In our analysis, HPCTP, PFPCTP, HCCTP, and HFCTP all demonstrated relatively high probabilities, ranging from 0.616 to 1. Thus, the combined results indicate that HPCTP, PFPCTP, HCCTP, and HFCTP may penetrate the BBB via passive diffusion.

**Table 3:** Molecular descriptors predicted by ADMETlab 3.0

Molecule	BBB	Molecular weight	Volume	nHA	nHD	logP	Caco-2 permeability	MDCK permeability	TPSA
HPCTP	0.616	693.13	669.074	9	0	3.284	−4.958	−4.709	92.46
PFPCTP	0.906	322.96	218.91	4	0	1.494	−4.969	−4.705	46.31
HCCTP	1	344.74	183.738	3	0	1.208	−5.03	−4.733	37.08
HFCTP	0.974	248.92	128.877	3	0	0.488	−5.073	−4.734	37.08
EPFCTP	0.215	274.96	166.192	4	0	0.745	−4.876	−4.688	46.31
HMCTP	0	321.04	248.989	9	0	−0.564	−4.842	−4.726	92.46

Nevertheless, it remains unclear whether CTPs and their *in vivo* metabolites may elicit more pronounced BBB toxicity and further affect the nervous system, which warrants further experimental investigation.

### BBB penetrating mechanisms of CTPs

#### Transporter function at the BBB

The BBB is formed by cerebral capillary endothelial cells, which exhibit specialized physiological functions that support an active transport system rich in carrier proteins. This system includes membrane proteins encoded by the ATP-binding cassette (ABC) transporter gene family, which primarily function to hydrolyze ATP and efflux xenobiotics or drugs from the CNS or cerebral endothelial cells, thereby providing neuroprotection and detoxification.^23^^,^^24^^,^^31^^,^^32^^,^^33^^,^^34^ Another critical group of BBB transporters comprises membrane-bound proteins encoded by the solute carrier (SLC) gene family that mediate bidirectional transport of various substances such as carbohydrates, amino acids, hormones, and fatty acids, between the bloodstream and the CNS.^23^^,^^31^

The sophisticated transporter network within the BBB offers CTPs potential pathways beyond passive diffusion to enhance CNS penetration. These include carrier-mediated transport and modulation of transporter activity. Further investigation is needed to explore alternative mechanisms by which CTPs may cross the BBB. Network toxicology provides an advanced framework for elucidating toxicity targets and mechanisms of environmental contaminants. Therefore, to examine whether CTPs may affect transport functions throughout varies transporter systems, we performed a network toxicology analysis method.

#### HPCTP exhibits significant potential to disrupt substance transport across the BBB

**Figure 3:**
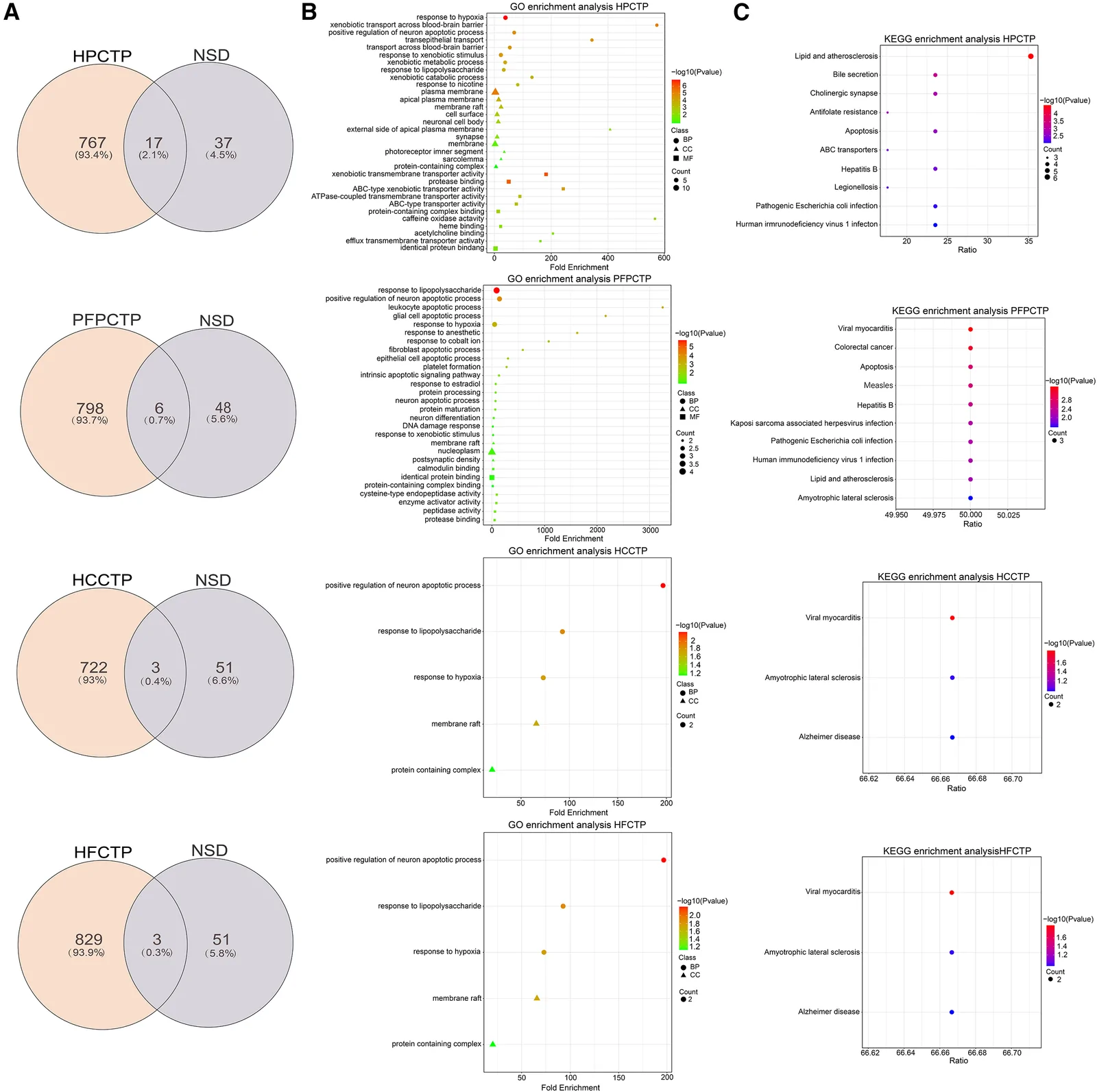
Differential gene analysis and functional enrichment of four CTP analogues (A) Venn diagrams illustrate the unique and shared differentially expressed genes (DEGs) induced by HPCTP, PFPCTP, HCCTP, and HFCTP relative to the NSD group, indicating compound-specific transcriptional responses. (B) GO enrichment analyses reveal that compound-specific DEGs are predominantly enriched in stress responses, programmed cell death, transmembrane transport, and metabolic regulation. (C) KEGG enrichment analyses show distinct pathway patterns among the four compounds, involving lipid metabolism regulation, apoptosis-related pathways, viral infection processes, and neurodegenerative disease pathways, suggesting multi-target toxicological mechanisms.

Based on prior screening outcomes, four compounds—HPCTP, PFPCTP, HCCTP, and HFCTP—were selected for network toxicology analysis. Potential targets were identified by querying each compound against Similarity Ensemble Approach (SEA), TargetNet, Index, SwissTargetPrediction, and the ChEMBL database. After removing duplicates, 784, 804, 725, and 832 target genes were obtained for HPCTP, PFPCTP, HCCTP, and HFCTP, respectively. These gene clusters were intersected with 54 high-confidence neurotoxicity-related genes associated with neurological system disorders (NSDs) from the Comparative Toxicogenomics Database (CTD), yielding 17, 6, 3, and 3 overlapping genes, respectively (Figure 3A). These overlapping genes are implicated in CNS and BBB functions.

The intersecting target genes of the four compounds were subsequently submitted to the DAVID database for Gene Ontology (GO) and Kyoto Encyclopedia of Genes and Genomes (KEGG) enrichment analyses. For HPCTP, GO analysis revealed significant enrichment in the biological process (BP) category for “response to hypoxia,” followed by “xenobiotic transport across BBB.” In the cellular component (CC) category, genes were enriched in “plasma membrane” and “apical plasma membrane.” Molecular function (MF) terms included “xenobiotic transmembrane transporter activity,” “protease binding,” and “ABC-type xenobiotic transporter activity” (Figure 3B). These findings suggest that HPCTP targets genes associated with the CNS and BBB, particularly localizing to plasma membrane structures and potentially influencing xenobiotic permeation and transporter functions on the BBB. For PFPCTP, HCCTP, and HFCTP, the intersecting target genes were primarily enriched in the BP terms “response to lipopolysaccharide” and “positive regulation of neuron apoptotic process,” with no significant enrichment observed in other categories (Figure 3B). This result suggests that PFPCTP, HCCTP, and HFCTP are potentially related to neuronal apoptosis.

KEGG pathway enrichment analysis indicated that the target genes of HPCTP were predominantly enriched in “lipid and atherosclerosis” pathways and exhibited associations with ABC transporters. Meanwhile, our data show that HPCTP and PFPCTP are associated with cell apoptosis, while HCCTP and HFCTP are related to Alzheimer disease (Figure 3C)).

These results imply that HPCTP is more likely than the other three compounds to modulate its own BBB permeability by interfering with multiple BPs, which include the expression of transporter genes, as well as the function and activity of transporters at the BBB. Moreover, HPCTP may enhance the penetration of other drugs and xenobiotics across the BBB.

#### Potential effects of CTPs in ABC efflux transporters

As indicated in Section transporter function at the BBB, the ABC efflux transporter family plays a critical role in eliminating xenobiotics from the CNS. Downregulation of ABC transporter gene expression or inhibition of their protein function may indirectly increase the retention of exogenous chemicals and drugs within the CNS.^35^ P-glycoprotein (P-gp/ABCB1), multidrug resistance-associated protein 1 (MRP1/ABCC1), and breast cancer resistance protein (BCRP/ABCG2) represent the principal ABC efflux transporters on the BBB.^23^^,^^36^^,^^37^

These proteins are expressed on the luminal membrane of cerebral capillaries, and P-gp is the predominant efflux pump at the BBB; therefore, P-gp is taken as the representative example.^33^ Given their function in transporting both endogenous and exogenous toxic substances and drugs from the CNS, a chemical identified as a P-gp substrate generally exhibits lower actual BBB penetration than predicted by its lipophilicity alone. Conversely, substances that inhibit P-gp may display enhanced BBB penetration due to reduced efflux activity, whereas increased drug lipophilicity on the BBB elevates the likelihood of a compound serving as a substrate for ABC efflux transporters.

As ABC transporters act as efflux pumps on the BBB, we speculate that the six CTPs may act both as P-gp substrates subject to efflux and as inhibitors that suppress P-gp activity, thereby reducing efflux and enhancing their likelihood of crossing the BBB. In our study, the ADMETlab 3.0 database was employed to predict the probability of the six CTPs serving as P-gp inhibitors, P-gp substrates, MRP1 inhibitors, or BCRP inhibitors. Results indicated that five CTPs (excluding HCCTP) showed high probabilities of acting as P-gp inhibitors (0.994–1.000). In contrast, all six CTPs exhibited negligible or absent potential as P-gp substrates (0–0.002). Regarding MRP1 inhibition, HPCTP demonstrated relatively low probability (0.195), whereas the other five CTPs displayed high probabilities (0.885–0.923). The above results were compiled and integrated to produce Table 4. The potential for BCRP inhibition was minimal across all six compounds. Therefore, all six compounds likely influence efflux transporters and may lead to their accumulation in the CNS, which is related to two potential mechanisms: inhibiting the function of specific ABC efflux transporters, or not being recognized as substrates by these transporters.

**Table 4:** Predicted probabilities of the six CTPs acting as ABC-transporter substrates or inhibitors

Molecule	Pgp-inhibitor	Pgp-substrate	MRP1 inhibitor	BCRP inhibitor
HPCTP	1	0	0.195	0
PFPCTP	0.998	0.001	0.885	0.001
HCCTP	0	0	0.913	0
HFCTP	0.994	0.002	0.895	0.001
EPFCTP	0.999	0	0.923	0
HMCTP	1	0	1	0.002

**Figure 4:**
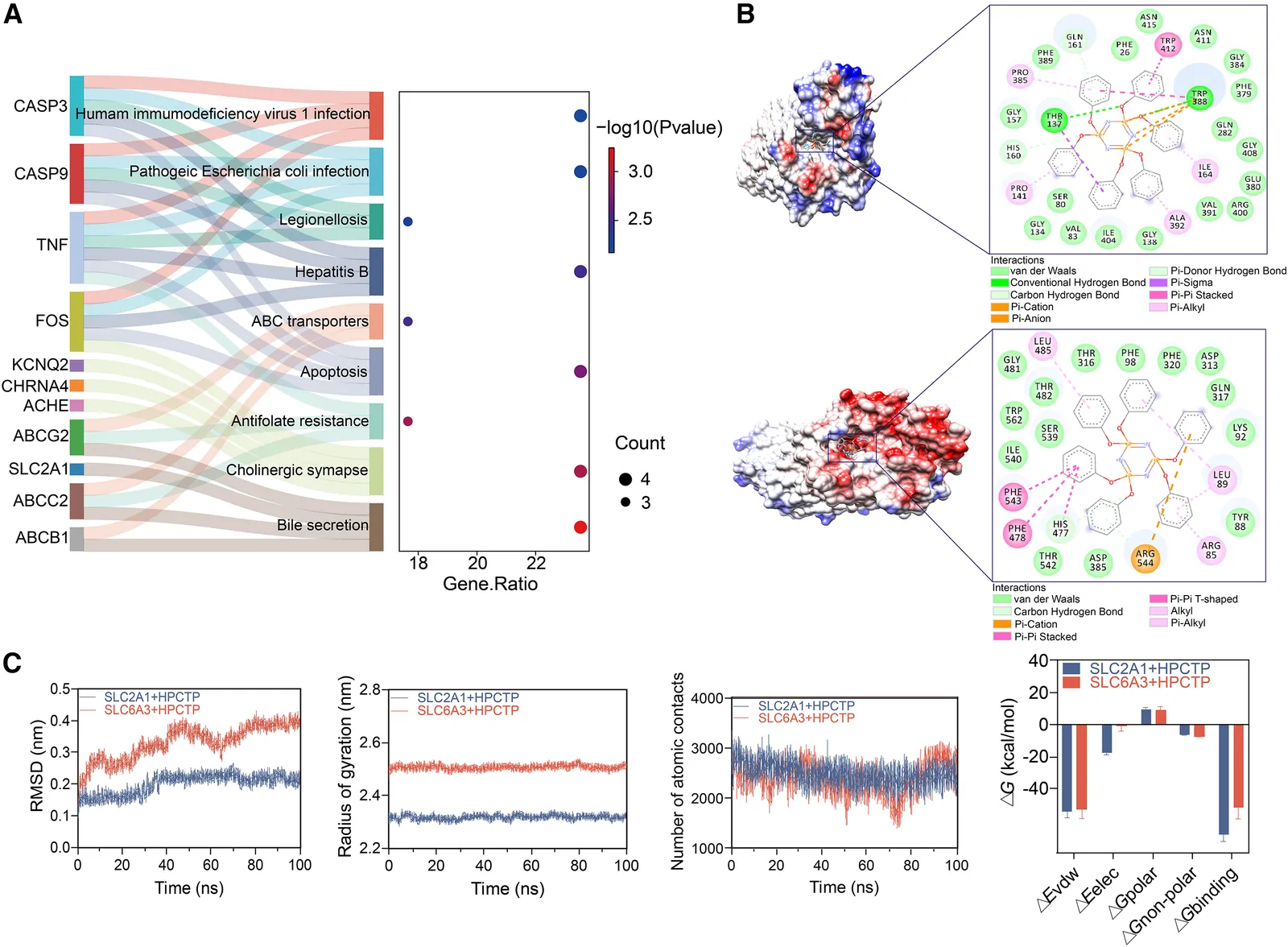
Pathway enrichment, molecular docking, and molecular dynamics analysis of HPCTP (A) Sankey and bubble plots summarize KEGG pathway enrichment of HPCTP-associated targets, highlighting significant involvement in apoptosis, ABC transporters, viral infection pathways, and cholinergic synapses. (B) Docking models of HPCTP with two representative proteins, identify key hydrogen bonds, hydrophobic contacts, and π-interactions contributing to ligand stabilization. (C) Molecular dynamics simulations indicate stable Rg trajectories for both HPCTP-SLC2A1 and HPCTP-SLC6A3 complexes, whereas the RMSD of HPCTP-SLC6A3 fluctuated markedly higher s; the SLC6A3 complex exhibits higher atomic contact counts and more favorable binding free energy, suggesting stronger interaction stability.

The preceding GO analysis indicated that HPCTP shows the strongest association with xenobiotic transport across the BBB. At the molecular function level, it is linked to xenobiotic transmembrane transporter activity and ABC-type transporter activity. We therefore hypothesize that HPCTP exerts a more pronounced influence on the ABC transporter system compared to other CTPs. Further examination has revealed that among the target genes related to CNS and BBB functions, HPCTP is associated with three ABC transporter family genes: *Abcb1* (*P-gp*), *Abcg2* (*Bcrp*), and *Abcc2* (*Mrp2*) (Figure 4A). These genes belong to the three major subfamilies responsible for efflux transport at the BBB.

Combined with the previous predictive results, these findings suggest that HPCTP may modulate ABC transporter function not only through direct inhibition of their activity, but also by altering their gene expression. Thus, different evaluations revealed that HPCTP exhibited a more extensive range of toxicity compared to the other five CTPs. Such effects could potentially lead to HPCTP-induced damage to the BBB barrier function and further alter efflux of other xenobiotics in the CNS.

#### HPCTP disrupts BBB SLCs

Combined literature analysis with network toxicology prediction revealed HPCTP’s potential association with SLC2A1 and SLC6A3 SLCs on the BBB, which suggests an alternative pathway for BBB penetration via transporter density and activity. Specifically, SLC2A1 (GLUT1) localizes to both luminal and abluminal membranes of brain capillary endothelial cells, primarily facilitating glucose and hexose transport from blood to brain, whereas SLC6A3 (DAT) functions as a neurotransmitter transporter with dopamine as its primary substrate.^38^^,^^39^

To evaluate the potential carrier-mediated transport of HPCTP across the BBB, we performed molecular docking simulations with SLC2A1 and SLC6A3. The computational binding affinities for HPCTP with SLC2A1 and SLC6A3 were—10.71 and—9.68 kcal/mol, respectively, both values significantly below the −5 kcal/mol threshold, indicating strong spontaneous binding. Detailed interaction analysis of HPCTP revealed an extensive network of molecular contacts in the SLC2A1 binding pocket, and intermolecular interaction (chemical bonds) as shown in the Figure 4B. Similarly, the SLC6A3 binding pocket accommodated HPCTP through chemical bonds in the Figure 4B.

Collectively, these computational analyses indicate that HPCTP exhibits favorable binding affinity and stable interactions with both SLC2A1 and SLC6A3 that are members of the SLC transporter family, and potentially influencing transporter-related processes at the BBB. However, these observations are based on computational predictions, further studies employing dedicated BBB permeability assays and *in vivo* models are required to validate these predictions.

#### Stable binding of HPCTP with two SLC transporters

Molecular dynamics simulations provide enhanced resolution of molecular interactions and transport dynamics facilitated by specific BBB transporters.^38^^,^^40^ We conducted all-atom molecular dynamics simulations spanning 100 ns to characterize the binding stability of HPCTP-SLC2A1 and HPCTP-SLC6A3. Results show that the SLC2A1-HPCTP complex maintained consistently low root-mean-square deviation (RMSD) values throughout the simulation, and rapidly reached equilibrium with minimal structural fluctuations, indicating constrained conformational flexibility and improved structural integrity (Figure 4C).

In comparison, the SLC6A3-HPCTP complex displayed significantly higher RMSD amplitudes with marked oscillations. These dynamics suggest more frequent conformational rearrangements and comparatively reduced stability. Both molecular complexes demonstrated stable radius of gyration trajectories during the entire simulation period (Figure 4C). This consistency implies that HPCTP binding induces compact structural organization in both receptor proteins.

Interfacial contact analysis provided additional insights, showing that SLC2A1 maintained substantially more atomic contacts with HPCTP than SLC6A3. The mean contact values were 2,504.2 ± 184.9 for SLC2A1 compared to 2,419.9 ± 256.2 for SLC6A3 (Figure 4C). These quantitative distinctions indicate a larger buried surface area and more robust non-covalent interactions in the SLC2A1-HPCTP complex. Conversely, the SLC6A3 interface showed fewer contacts with greater variability, reflecting weaker molecular engagement.

Binding free energy decomposition using the MM/PBSA method revealed that complex stabilization primarily resulted from favorable van der Waals interactions and nonpolar solvation terms, while polar solvation effects partially counterbalanced electrostatic contributions. The SLC2A1-HPCTP complex demonstrated more favorable values for both van der Waals and nonpolar solvation components. This energetic profile translated to stronger overall binding affinity, as evidenced by more negative ΔGbinding values compared to the SLC6A3-HPCTP system (Figure 4C). The observed energetic advantage for SLC2A1 aligns with its lower RMSD fluctuations and higher contact density. Together, these computational findings consistently support the superior binding stability of HPCTP with SLC2A1 compared to SLC6A3.

**Figure 5:**
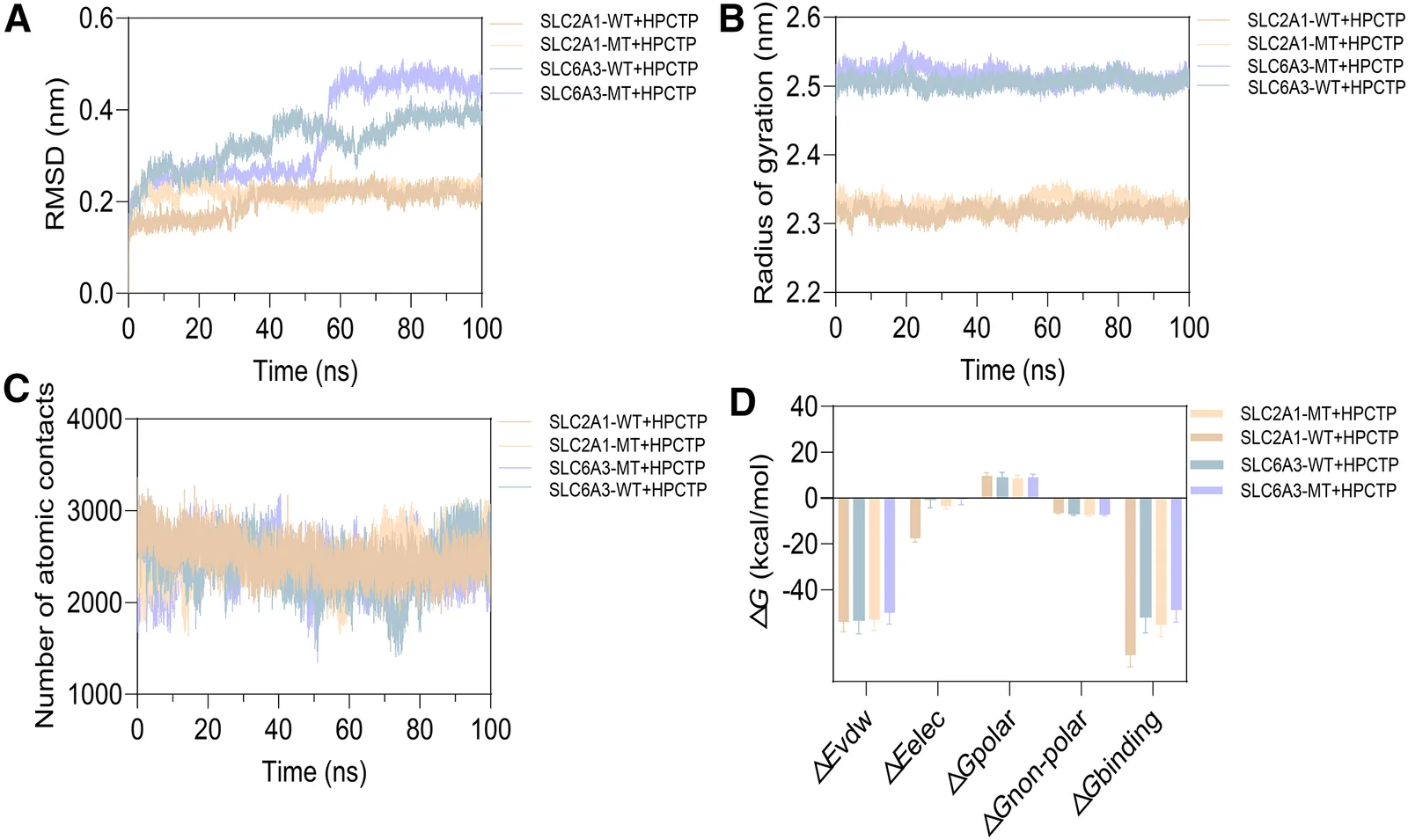
Computational identification of key HPCTP-binding residues in SLC2A1 and SLC6A3

Moreover, RMSD analysis revealed that after the substitution of the key residues with alanine, HPCTP still bound to SLC2A1 and SLC6A3, but the mutant complexes exhibited lower number of atomic contacts and slightly increased radius of gyration, indicating a looser packing of the binding interface (Figures 5A–5C). Consistently, the binding free energies became less favorable in the mutant systems, further demonstrating that mutation of these key residues weakens the stability and affinity of HPCTP binding to both transporters (Figure 5D). These observations confirm that the interaction pattern predicted by our molecular docking, in which these residues play a central role in HPCTP recognition, is reliable.

#### HPCTP exposure affects the glucose uptake of hCMEC/D3 cells

**Figure 6:**
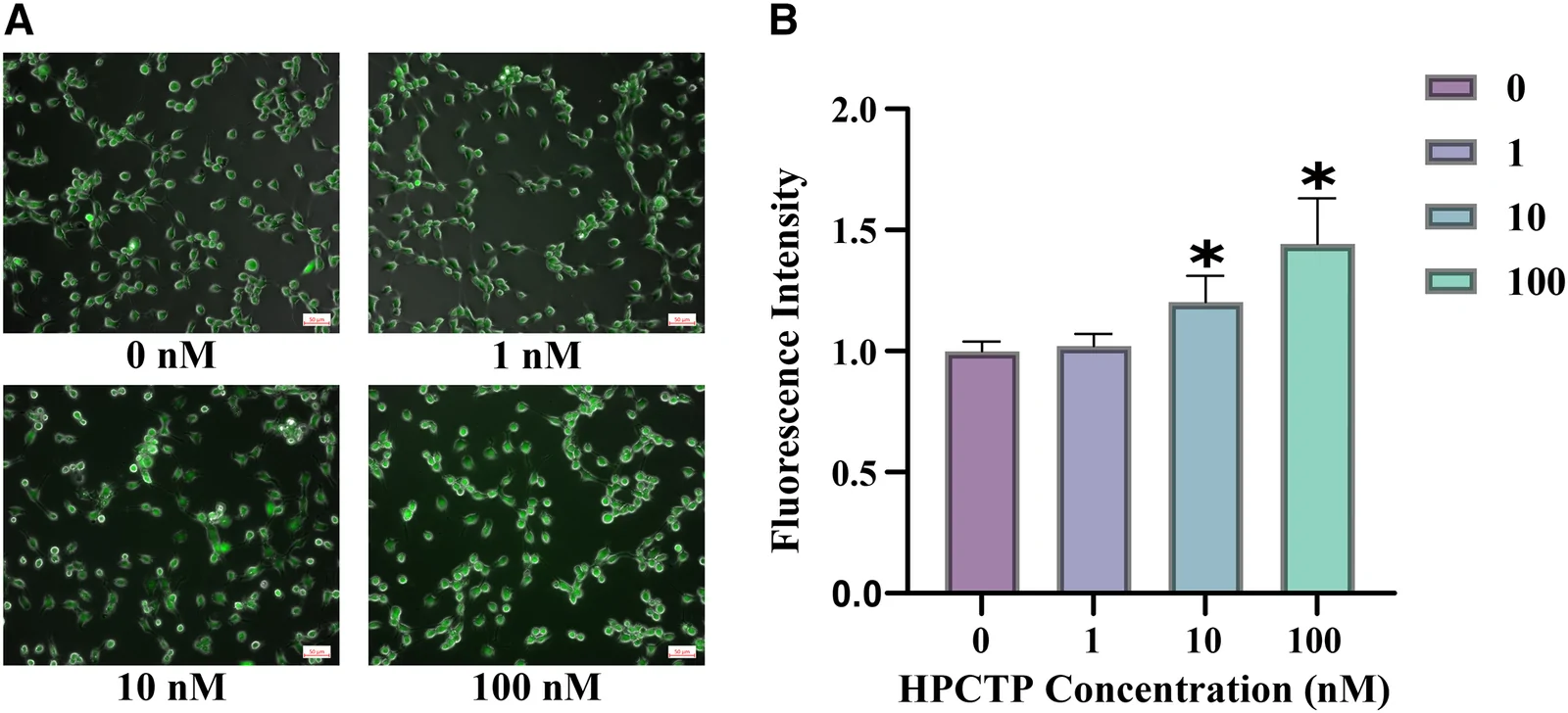
Effects of HPCTP exposure on glucose uptake in hCMEC/D3 cells (A) Representative fluorescence images of intracellular glucose uptake in hCMEC/D3 cells following exposure to different concentrations of HPCTP. Green fluorescence indicates intracellular glucose accumulation. Scale bars, 50 μm. (B) Quantitative analysis of fluorescence intensity in hCMEC/D3 cells after HPCTP exposure. ∗*p* < 0.05, *n* = 3, data are presented as mean ± SEM.

We observed a dose-dependent increase in fluorescence intensity in hCMEC/D3 cells following exposure to HPCTP at concentrations of 0, 1, 10, and 100 nM (Figure 6A). Statistical analysis demonstrated that compared with the control group, the fluorescence intensities in the 1, 10, and 100 nM groups increased significantly with increasing concentration (Figure 6B). These findings suggest that HPCTP exposure may alter the glucose uptake capacity of hCMEC/D3 cells.

#### HPCTP exposure affected the glucose transport, metabolism and *Slc2a1* gene expression in hCMEC/D3 cells

**Figure 7:**
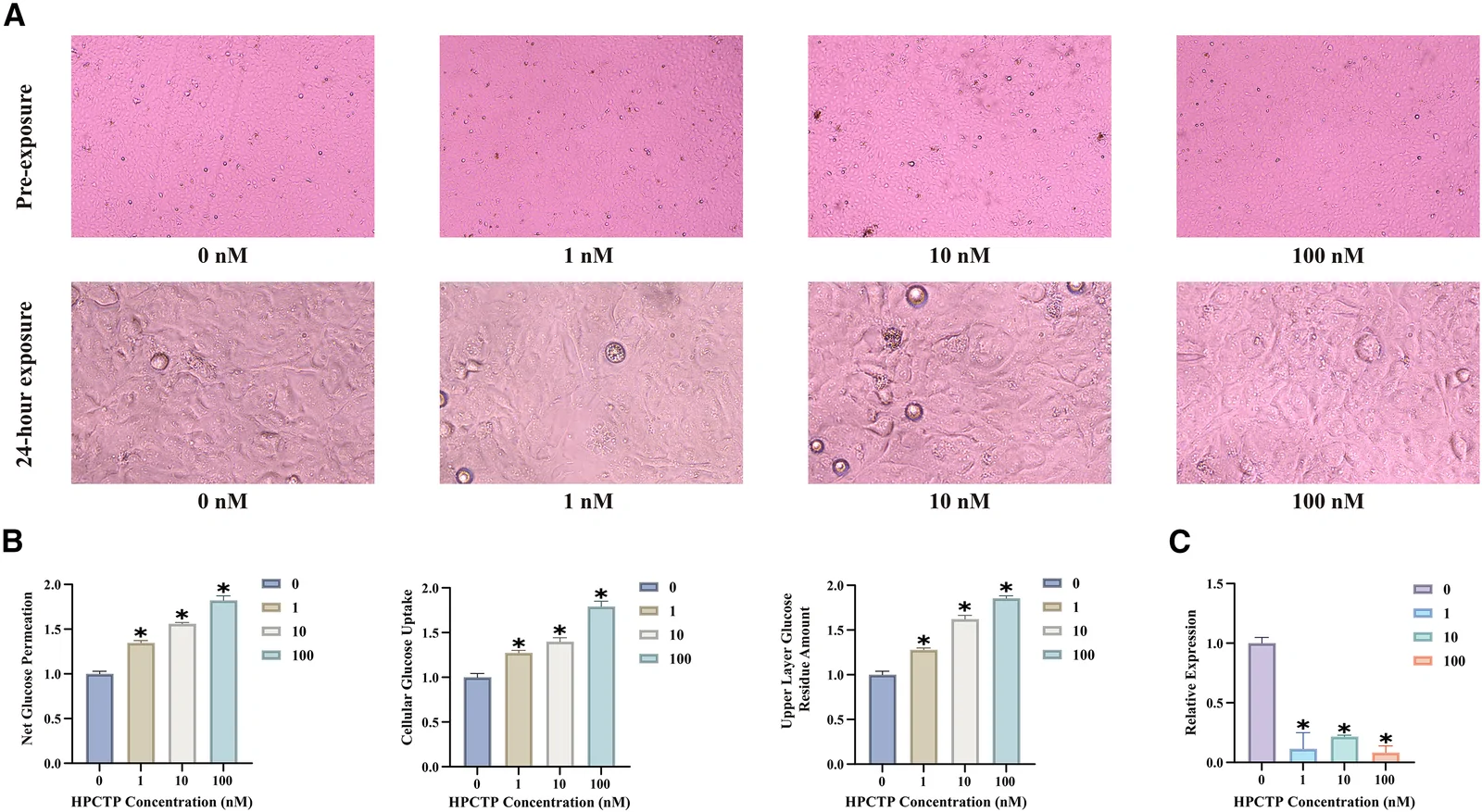
Morphological observation, quantitative analysis of cells under different treatment conditions, and RT-qPCR statistical results of cells after exposure (A) Bright-field microscopy observation of cell morphology in the control group (×10 magnification, scale bars, 100 μm) and experimental group (×20 magnification, scale bars, 50 μm). (B) Corresponding quantitative statistical analysis of net glucose permeation, cellular glucose uptake and upper layer glucose residue amount. (C) Relative expression of *Slc2a1* gene in hCMEC/D3 cells. ∗*p* < 0.05, *n* = 3, data are presented as mean ± SEM.

To further investigate the effects of HPCTP exposure on glucose transport across the BBB, we established a compact hCMEC/D3 cell monolayer to simulate the BBB for permeability assays. According to the light microscope images, before and after the exposure, there were no changes in the cell monolayer membranes, and the connections between cells were not damaged after the exposure (Figure 7A). Compared with the control group, the glucose concentration in the lower chamber, cell lysates, and upper chamber increased significantly in a dose-dependent manner. Since consumed glucose is primarily metabolized by cells, the observed increases in glucose concentrations suggest that HPCTP exposure may inhibit glucose utilization in hCMEC/D3 cells. Moreover, the dose-dependent increase in glucose concentration in the upper chamber medium indicates that HPCTP exposure may impair the transmembrane transport capacity of hCMEC/D3 cells for glucose (Figure 7B). Interestingly, HPCTP exposure significantly downregulated the expression of the *Slc2a1* gene in hCMEC/D3 cells. Given the critical role of SLC2A1 in BBB glucose transport, this effect may represent an important mechanism by which HPCTP inhibits glucose transport across the BBB.

#### Overview of predicted HPCTP BBB penetration

Based on our previous predictions, we propose that HPCTP may cross BBB through both passive diffusion and SLC-mediated transport, and may also function as an inhibitor of ABC transporters. SLC2A1 is abundantly expressed on both the luminal and abluminal membranes of cerebral capillary endothelial cells, and molecular docking analyses indicate favorable binding between HPCTP and SLC2A1. Accordingly, we speculate that HPCTP facilitates its own BBB penetration by interacting with the SLC2A1 transporter.

**Figure 8:**
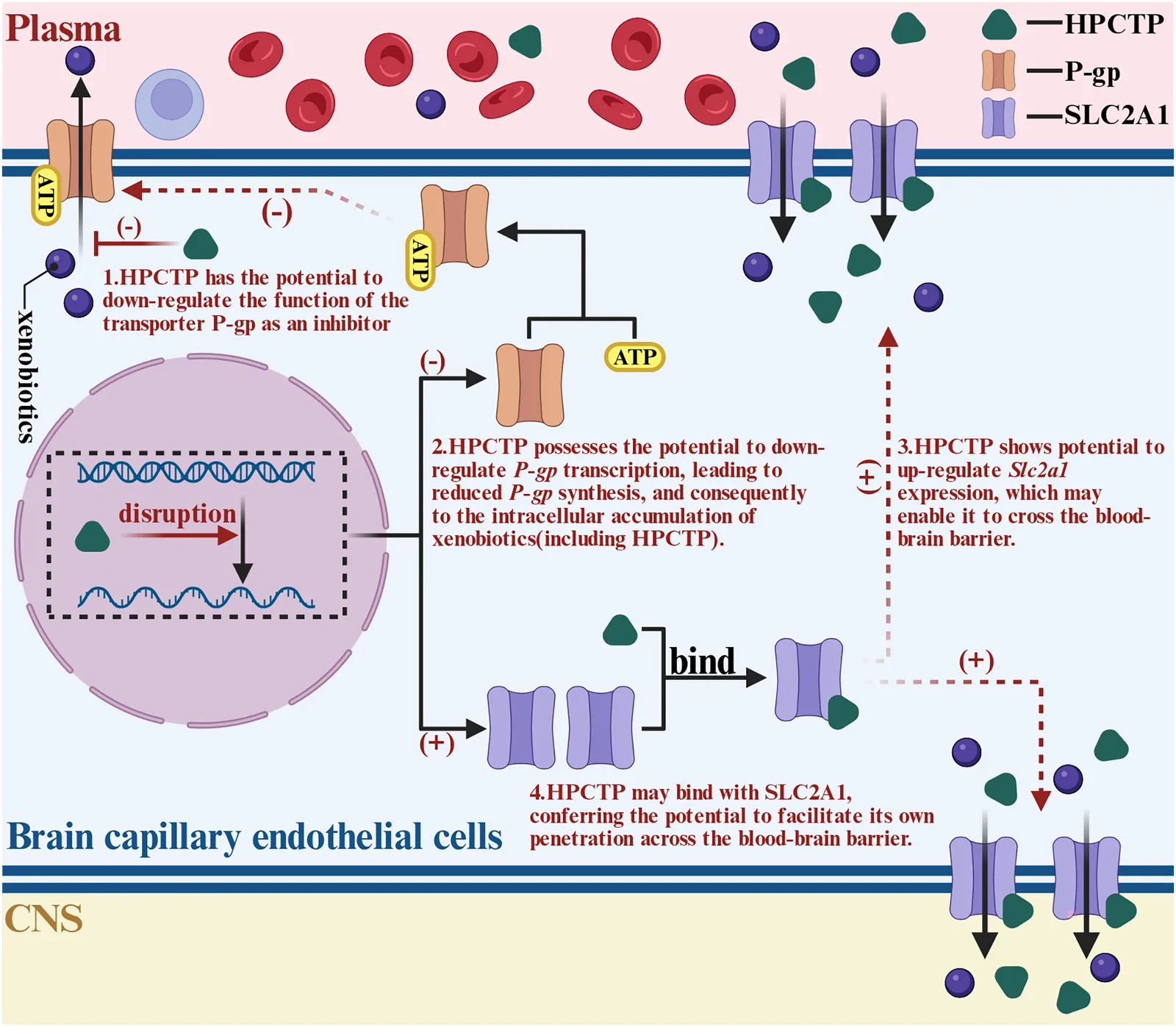
Molecular mechanism of HPCTP penetrating the BBB

Network-toxicology GO enrichment analysis further suggests that HPCTP regulates the expression of genes within the SLC and ABC transporter families. Specifically, HPCTP may upregulate *Slc2a1*, thereby increasing the density of SLC2A1 transporters on endothelial surfaces and enhancing its own translocation across the BBB. Conversely, HPCTP may downregulate the ABC efflux genes *Abcb1* (*P-gp*), *Abcg2* (*Bcrp*), and *Abcc2* (*Mrp2*), reducing the expression of the major ABC efflux subfamilies and consequently increasing intracellular HPCTP levels. In addition, ADMETlab 3.0 predicts that HPCTP acts as a P-gp inhibitor, further elevating endothelial HPCTP accumulation and increasing the likelihood of successful BBB penetration (Figure 8).

## Discussion

The BBB permeability of chemicals is of crucial importance for the assessment of neurotoxicity; therefore, we innovatively constructed an assessment method for BBB permeability by computational toxicity and physicochemical property indicators. Using machine learning and QSAR modeling, we found that all six CTPs may alter the permeability of the BBB due to their effects on its structure and function. Among CTPs, HPCTP showed the strongest BBB toxicity. Further analysis confirmed that all six CTPs possess physicochemical characteristics supporting passive diffusion across the BBB. We then applied network toxicology, molecular docking, and molecular dynamics simulations to identify other penetration routes. Previous study has been demonstrated that increased BBB permeability facilitated the transport of exogenous substances into the brain, highlighting the importance of BBB integrity in controlling CNS exposure.^41^

The demand for high-performance lithium-ion batteries continues to grow, especially in the electric vehicle sector, and this trend has significantly increased the production and use of CTPs.^1^^,^^2^ Recent work revealed that HPCTP disrupts several neurodevelopment-related genes expression such as *Mbpa*, *Syn2a*, *Gap43*, and *Gfap*, resulting in the impairment of neuronal differentiation, synaptogenesis, and glial maturation, and also downregulates *Htr1aa* expression, suppressing 5-HT1A receptor function and further inducing depression-like behavior in experimental models.^18^^,^^20^^,^^21^ However, these studies do not seem to explain the scientific issue of how HPCTP penetrates the BBB. Previous studies have established a correlation between the likelihood of passive diffusion across the BBB and specific physicochemical properties of molecules.^24^^,^^42^^,^^43^ These properties have been utilized in screening strategies for CNS-targeting drugs, such as in evaluating the BBB penetration potential of phenyl-γ-valerolactones (PVLs) and phenylvaleric acids (PVAs).^25^^,^^44^ Thus, our study conducted a BBB toxicity assessment of CTPs via implementing a scoring system based on molecular descriptors derived from the QikProp module in Schrödinger software. This analysis confirms that six CTP analogues can penetrate the BBB via passive diffusion and identifies HPCTP, PFPCTP, HCCTP, and HFCTP as having significant potential for penetrating and damaging the BBB.

To further investigate the molecular mechanism by which HPCTP, PFPCTP, HCCTP, and HFCTP penetrate the BBB, we applied network toxicology methods performing GO and KEGG analyses on their CNS- and BBB-related target genes. Results show that four CTP analogues are linked to the CNS via GO enrichment analysis. More critically, HPCTP exhibits stronger associations than the other three compounds across BP, CC, and MF modules, which is similar to previous study showing HPCTP poses greater potential hazards to living organisms than other CTP analogues.^17^^,^^18^^,^^20^ And moreover, HPCTP is clearly related to the ABC efflux transporters and SLC transporters on the BBB, suggesting its potential to modulate key nodes governing BBB permeability. Therefore, our analysis focuses on HPCTP, exploring the active transport of HPCTP by ABC and SLC transporters.

For ABC efflux transporters, target gene prediction revealed that HPCTP associates with genes encoding *P-gp*/*Abcb1*, *Mrp1*/*Abcc1*, and *Bcrp*/*Abcg2*, implying potential disruption of their expression. In addition, predictions from ADMETlab 3.0 suggested that there is a high probability that HPCTP is an inhibitor of P-gp rather than a substrate, and we also discovered that HPCTP has low probability of inhibiting MRP1 and is unlikely to act as a BCRP inhibitor. In general, if a chemical substance meets conditions of downregulating the expression of efflux transporter genes on the BBB, inhibiting their activity and not acting as their substrate, then chemical substance will be less transferred from CNS to bloodstream by efflux transporter system, which may lead to a greater accumulation of chemical substance in the CNS.^45^ Therefore, we do not exclude the possibility that HPCTP inhibits the P-gp transporter located on the BBB, thereby accumulating more harmful substances in the brain during mixed exposure.

Analysis of HPCTP’s interactions with SLC transporters identified *Slc2a1* and *Slc6a3* as potential target genes, indicating its capacity to interfere with SLC function and subsequently influence BBB permeability to xenobiotics. Thus, we hypothesize that HPCTP perhaps acts as a substrate for SLC2A1 and SLC6A3 facilitating its own BBB penetration. Subsequently, molecular docking and dynamics simulation results demonstrated strong binding affinity and stability for both HPCTP-SLC2A1 and HPCTP-SLC6A3 complexes, supporting the possibility of HPCTP serving as a substrate for SLC transporters.

Importantly, molecular dynamics simulations further revealed distinct interaction patterns between HPCTP and the two SLC transporters. As shown in Figure 4C, the SLC2A1-HPCTP complex maintained substantially lower RMSD values throughout the 100 ns simulation compared with the SLC6A3-HPCTP complex, indicating higher conformational stability of the SLC2A1-bound system. Similarly, the radius of gyration (Rg) of the SLC2A1-HPCTP complex remained relatively stable during the simulation, whereas the SLC6A3-HPCTP complex exhibited greater structural fluctuations. In addition, the SLC2A1-HPCTP system consistently maintained a higher number of atomic contacts, suggesting tighter and more persistent intermolecular interactions between HPCTP and SLC2A1. Binding free energy analysis further supported these observations. The total binding free energy (ΔGbinding) of the SLC2A1-HPCTP complex was lower than that of the SLC6A3-HPCTP complex, indicating a thermodynamically more favorable interaction. Notably, van der Waals interactions and electrostatic contributions represented the major driving forces stabilizing the SLC2A1-HPCTP complex. Molecular dynamics simulations following virtual point mutations confirmed that the interaction patterns predicted by our molecular docking analysis were reliable. These results collectively suggest that HPCTP may exhibit a stronger interaction tendency toward SLC2A1 than SLC6A3.

Considering that SLC2A1 (GLUT1) is the principal glucose transporter expressed at the BBB and is essential for maintaining cerebral glucose supply, the stronger and more stable interaction between HPCTP and SLC2A1 may have important biological implications beyond BBB penetration. To further validate the biological relevance of the computational predictions, we subsequently performed *in vitro* experiments using the hCMEC/D3 cells to simulate the BBB. Interestingly, HPCTP exposure induced dose-dependent alterations in glucose uptake, glucose permeability, and intracellular glucose accumulation, accompanied by significant downregulation of *Slc2a1* gene expression, which was consistent with the mechanism predicted in our network toxicology analysis. This phenomenon may be associated with the dual effects of HPCTP on glucose uptake and glucose metabolism in hCMEC/D3 cells.

Moreover, the total glucose concentration in the entire system, including the upper chamber medium, lower chamber medium, and cell lysates, also increased in a dose-dependent manner. These findings indicate that HPCTP may interfere with the normal glucose transport function of SLC2A1, thereby disrupting BBB energy metabolism as well as influencing energy supply and metabolic utilization between the BBB and the CNS. Previous studies have demonstrated that BBB dysfunction and alterations in cerebral oxygen and nutrient delivery are closely associated with brain injury progression and neurological impairment.^46^ Therefore, disruption of SLC2A1-mediated glucose transport by HPCTP may represent an important mechanism contributing to BBB dysfunction and CNS toxicity.

Previous studies have shown that reduced GLUT1 expression at the BBB is associated with BBB breakdown, impaired cerebral glucose uptake, which is linked to neurodegeneration in Alzheimer disease.^47^ In addition, GLUT1 dysfunction has also been implicated in neurological disorders such as epilepsy due to impaired cerebral energy metabolism.^48^ Therefore, HPCTP-induced disturbance of SLC2A1 may indicate a potential risk for BBB dysfunction and neurological impairment.

In summary, this study utilized computational toxicology methods to establish a reliable assessment system to evaluate the BBB permeability of CTPs, revealing distinct mechanisms by which CTPs may penetrate the BBB, including passive diffusion and transporter-mediated penetrating. Importantly, our research further demonstrated that HPCTP exposure exerted adverse effects on glucose transport and metabolism, suggesting its potential risk in disrupting CNS energy metabolism. Based on our findings, we proposed that CTP-induced effects on the BBB potentially alter its permeability to other substances. Our research findings underscore the necessity of conducting more in-depth toxicological studies on CTPs, particularly regarding their neurotoxicity. Given the relevance of these compounds to human and environmental health, more *in vitro* and *in vivo* studies are essential to fully characterize their risk profiles.

### Limitations of the study

The present study has certain limitations, although our *in vitro* experiments demonstrated that HPCTP affects glucose uptake, glucose distribution, and *Slc2a1* expression in hCMEC/D3 cells, our research did not directly demonstrate BBB penetration by HPCTP or its direct impact on BBB integrity. Therefore, further studies employing dedicated BBB permeability assays and *in vivo* models are required to validate these predictions and clarify the effects of HPCTP on BBB function and permeability.

## Resource availability

### Lead contact

Request for further information, resources, and reagents should be directed to and will be fulfilled by the lead contact, Jiaqi Zhou (zjqdk@foxmail.com).

### Materials availability

This study did not generate any new unique reagents. All chemicals, biological materials, and commercial reagents used in this study are commercially available and were obtained from the indicated suppliers described in the STAR Methods section.

### Data and code availability

All data generated or analyzed during this study are included in this article and its supplemental information. Additional raw data supporting the findings of this study are available from the corresponding authors upon reasonable request. No original code was developed for this study; all computational analyses were performed using publicly available software and online databases as described in the STAR Methods section.

## Acknowledgments

This research was supported by the 10.13039/501100010617Dali University (KY2396120340) and the 10.13039/501100007846Yunnan Provincial Department of Education (KY2414107040) (S202510679099). The illustrations were created with BioRender.

## Author contributions

Q.Y., methodology, writing – original draft, validation, and data curation; J.Z., writing – review and editing, investigation, and software; Y.D., resources and conceptualization; Z.H., data curation and investigation; R.L., formal analysis and conceptualization; R.J., methodology and formal analysis; Y.W., software and investigation; S.L., formal analysis; C.Z., data curation; Z.Z., visualization and formal analysis; J.Z., supervision, project administration, and funding acquisition.

## Declaration of interests

The authors declare no competing interests.

## STAR★Methods

### Key resources table

**Table:** 

REAGENT or RESOURCE	SOURCE	IDENTIFIER
**Chemicals, peptides, and recombinant proteins**		

Hexaphenoxycyclotriphosphazene (HPCTP)	Shanghai Macklin Biochemical Technology Co., Ltd. (Shanghai, China)	CAS: 1184-10-7
DMSO (cell culture grade)	Dalian Meilun Biotechnology Co., Ltd. (Dalian, China)	Cat.No.: PWL064
PBS buffer (1×)	Wuhan Pricella Biotechnology Co., Ltd. (Wuhan, China).	Cat.No.: PB180327
0.25% Trypsin solution	Wuhan Pricella Biotechnology Co., Ltd. (Wuhan, China).	Cat.No.: PB180225
Click Reaction Buffer	Shanghai Beyotime Biotechnology Co., Ltd. (Shanghai, China)	Cat.No.: C0071S

**Critical commercial assays**		

Glucose uptake assay kit (2-NBDG fluorescence method)	Wuhan Servicebio Technology Co., Ltd. (Wuhan, China)	Cat.No.: G1744-200UL
Animal Tissue/Cell Total RNA Extraction Kit	Wuhan Servicebio Technology Co., Ltd. (Wuhan, China)	Cat.No.: G3640-50T
Cell RNA Extraction Kit	Absin Bioscience Inc. (Shanghai, China).	Cat.No.: abs60286-50T
Total RNA Extraction Reagent	Absin Bioscience Inc. (Shanghai, China).	Cat.No.: abs60154
SYBR Advanced qPCR SuperMix Kit	Absin Bioscience Inc. (Shanghai, China).	Cat.No.: abs60087-500T
One-step reverse transcription third-generation premix	Absin Bioscience Inc. (Shanghai, China).	Cat.No.: abs60246-100T
Antifade Mounting Medium	Coolaber Science & Technology Co., Ltd. (Beijing, China)	Cat.No.: SL1841

**Deposited data**		

SEA (Similarity EnsembleApproach)	SEA Search Server	https://sea.bkslab.org/
TargetNet	TargetNet server	http://targetnet.scbdd.com
SwissTargetPrediction	Swiss Institute of Bioinformatics	http://www.swisstargetprediction.ch
ChEMBL database	EMBL-EBI	https://www.ebi.ac.uk/chembl/
Comparative ToxicogenomicsDatabase (CTD)	Comparative Toxicogenomics Database	https://ctdbase.org/
DAVID Bioinformatics Resources	Laboratory of Human Retrovirology and Immunoinformatics	https://davidbioinformatics.nih.gov/
Index	Index database	http://targetnet.scbdd.com/calcnet/index/
Protein DataBank (PDB)	RCSB Protein DataBank	https://www.pdbus.org/
PubChem	National Center for Biotechnology Information (NCBI)	https://pubchem.ncbi.nlm.nih.gov/

**Experimental models: Cell lines**		

hCMEC/D3 complete culture medium	Zhejiang Meisen Cell Technology Co., Ltd. (Zhejiang, China)	Cat.No.: CTCC-003-0113-CM

**Software and algorithms**		

ProTox 3.0	Charité – Universitätsmedizin Berlin	https://tox.charite.de/protox3/
SWISS-MODEL	SWISS-MODEL	https://swissmodel.expasy.org/
ADMETlab 3.0	Computational Biology and Drug Design Group, Xiangya School of Pharmaceutical Sciences	https://admetlab3.scbdd.com/
Maestro software	Schrödinger, LLC	Version 2024-4
Linear Constraint Solver (LINCS) algorithm.	GROMACS development team	GROMACS built-in algorithm
Particle-mesh Ewald (PME) method	GROMACS development team	GROMACS built-in algorithm
MM - GBSA method	Computational method	N/A
gmx_MMPBSA	gmx_MMPBSA developers	https://github.com/Valdes-Tresanco-MS/gmx_MMPBSA
GROMACS 5.1.4 software	GROMACS development team	Version 5.1.4
AutoDock 4.2.6	The Scripps Research Institute	Version 4.2.6
AutoDockTools 1.5.6.	The Scripps Research Institute	Version 1.5.6

**Other**		

PCR plates	Wuhan Servicebio Technology Co., Ltd. (Wuhan, China)	Cat.No.: PCR-9601W-HS

### Experimental model and study participant details

#### Cell culture model

Human cerebral microvascular endothelial cells (hCMEC/D3) and the corresponding complete culture medium were obtained from Zhejiang Meisen Cell Technology Co., Ltd. (Zhejiang, China). Cells were cultured under standard conditions (37°C, 5% CO_2_) and used as an *in vitro* BBB model.

#### Chemical exposure model

Hexaphenoxycyclotriphosphazene was purchased from Shanghai Macklin Biochemical Technology Co., Ltd. (Shanghai, China).

#### Computational model

We obtained SMILES notations and two-dimensional structures of the six CTP compounds from the PubChem database. Among these, PFPCTP, HCCTP, HFCTP, EPFCTP, and HMCTP had directly available three-dimensional structures. For HPCTP, which lacked a 3D structure in the database, we generated an initial 3D model from its 2D structure using Avogadro-1.2.0 software and performed geometry optimization.

### Method details

#### Establishment of criteria for assessing the potential of exogenous compounds to cross the blood - Brain barrier

Research has established that the passive diffusion of exogenous compounds across the blood-brain barrier (BBB) correlates strongly with specific physicochemical properties. Several early studies reported that physicochemical properties of compounds, including molecular weight, lipophilicity, hydrogen-bonding capacity, and polar surface area, were closely associated with their ability to cross the BBB via passive diffusion. These studies further explored the use of computationally predicted physicochemical parameters to preliminarily evaluate the passive BBB permeability of compounds. Meanwhile, the predicted permeability outcomes were initially compared with results obtained from *in vivo* animal experiments and *in vitro* cellular assays.^26^^,^^27^^,^^42^ Subsequent studies continuously expanded and refined the physicochemical descriptors associated with passive BBB diffusion. In addition, the validity and reliability of computational prediction approaches were further substantiated through comprehensive comparisons between predicted permeability results and experimental observations.^25^^,^^28^^,^^38^^,^^43^^,^^44^ Computational toxicology tools can reliably predict these properties currently. Available platforms include the QikProp module in Schrödinger software and ADMETlab 3.0.^28^^,^^42^^,^^43^^,^^44^

Latest literature lists the 12 computational physicochemical property parameters most relevant to the performance of compounds in passive diffusion through BBB, Based on this literature, we have built a scoring system to quantitatively assess BBB penetration possibility.^44^ This system incorporates 12 parameters obtained from Schrödinger software predictions. The selected parameters are: predicted BBB partition coefficient (QPlogBB), MDCK cell permeability, molecular weight, octanol/water partition coefficient (logP), dipole moment, molecular volume, hydrogen bond donor count, hydrogen bond acceptor count, Caco-2 cell permeability, predicted albumin binding affinity (logKhsa), polar surface area (PSA), and number of rotatable bonds. For each parameter, we identified literature-derived optimal ranges that favor passive BBB diffusion.^25^^,^^26^^,^^27^^,^^28^^,^^29^^,^^44^ Compounds earn one point for each parameter falling within its optimal range and the summation produces a composite BBB score ranging from 0 to 12 (Table 1).

#### Computer prediction of BBB permeability and toxicity for CTPs

Six CTP compounds molecular structures were converted to MOL format and imported into Maestro software (version 2024-4, Schrödinger) for subsequent processing. Structural processing employed the LigPrep module with the Optimized Potentials for Liquid Simulations (OPLS) force field. The optimized structures were then analyzed using the QikProp module, which generated 51 molecular descriptors for each compound. We selected 12 key parameters from this set for inclusion in our BBB penetration scoring system, as specified in Table 1.

The ADMETlab 3.0 platform, which implements machine learning and Quantitative Structure-Activity Relationship (QSAR) modeling, provided predictions for absorption, distribution, metabolism, excretion, and toxicity properties.^30^^,^^43^ We submitted SMILES notations of all six CTPs to this platform for analysis. From the resulting output parameters, we selected 10 descriptors for comparative analysis along with the 12 parameters obtained from QikProp predictions. The BBB index calculated by ADMETlab 3.0 served as an additional indicator for identifying compounds with enhanced BBB penetration potential.^43^^,^^44^^,^^49^ We next identified nomenclature inconsistencies between certain parameters shared by Schrödinger and ADMETlab 3.0 outputs. To resolve these discrepancies, we consulted official documentation from both platforms to establish accurate parameter correspondences and annotated these relationships accordingly. Additionally, we submitted the SMILES notations of all six CTPs into the ProTox 3.0 predictive toxicology platform. The resulting toxicity prediction radar charts provided supplementary data for our toxicity assessment.

#### Screening of potential target genes associated with CTPs and blood - Brain barrier function

Following initial screening, we employed five specialized databases to identify potential target genes for HPCTP, PFPCTP, HCCTP, and HFCTP. These databases included SEA, TargetNet, Index, SwissTargetPrediction, and ChEMBL. All searches were limited to Homo sapiens. We removed duplicate entries from the initial results and consolidated the remaining genes for each compound.

We then obtained high-confidence neurotoxicity-related genes from the Comparative Toxicogenomics Database (CTD). These genes were associated with neurological system disorders (NSD). The consolidated target genes from our database searches were cross-referenced with this neurotoxicity gene set. This intersection analysis identified overlapping target genes that may participate in both CTP exposure and neurological toxicity pathways.

#### Functional pathway analysis of target genes

The DAVID database provides an online bioinformatics resource for systematic functional annotation of gene and protein datasets. We employed this platform to analyze potential target genes related to blood-brain barrier (BBB) functions for the four selected CTPs. Our analysis included both Gene Ontology (GO) and Kyoto Encyclopedia of Genes and Genomes (KEGG) enrichment approaches. The GO examination encompassed three distinct categories: Biological Process (BP), Cellular Component (CC), and Molecular Function (MF). We established a statistical significance threshold of *p*-value <0.05 for identifying enriched functional terms. This analytical strategy helped clarify the biological significance and potential molecular mechanisms of the candidate genes.

#### Selection of core targets and molecular docking

Molecular docking (MD) simulates molecular interactions between ligands and biological targets. This computational method examines binding interactions with macromolecular proteins at the blood-brain barrier (BBB). The technique finds broad application in biomedical research, particularly in drug discovery and development. Combining molecular docking with other computational methods enhances prediction reliability and enables investigation of potential mechanisms for CTP penetration across the BBB.^38^^,^^50^

We identified potential solute carriers that interact with HPCTP by combining GO and KEGG enrichment results with established literature on BBB solute carriers. Three-dimensional structures of two solute carrier proteins, SLC2A1 (PDB ID: 6THA) and SLC6A3 (PDB ID: 9EO4), were obtained from the Protein DataBank. We removed crystallographic water molecules, heteroatoms, and native ligands from these structures, and missing residues and atoms were modeled using SWISS-MODEL. Protein preparation involved AutoDockTools 1.5.6. For both SLC2A1 and SLC6A3, we added hydrogen atoms, merged nonpolar hydrogens, and assigned Gasteiger partial charges. The prepared receptor structures were then converted to PDBQT format.^51^ For the HPCTP ligand, we added hydrogen atoms at physiological pH (7.(4) using Avogadro software. Subsequent processing in AutoDockTools involved merging nonpolar hydrogens and assigning partial charges before final format conversion.

Docking simulations employed AutoDock 4.2.6 with the Genetic Algorithm (GA). We conducted 100 independent runs for each docking experiment while maintaining default parameters for other settings. The resulting complex conformations underwent cluster analysis. We selected the highest-scoring representative conformation from each cluster for detailed interaction pattern examination.^51^

#### Molecular dynamics simulations

Molecular dynamics simulations offer detailed understanding of molecular interactions during chemical transport across the blood-brain barrier. These simulations help predict permeability involving active transport mechanisms.^38^ We conducted molecular dynamics simulations to evaluate binding stability between HPCTP and two solute carriers, SLC2A1 and SLC6A3. The simulations used GROMACS 5.1.4 software with the Amber ff99SB force field.^52^^,^^53^

We then generated force field parameters for HPCTP using the general Amber force field through AmberTools20. The complex system underwent solvation in a periodic dodecahedral box with the TIP3P water model. A minimum distance of 1.2 nm was maintained between solute atoms and box boundaries. The system was supplemented with 150 mM NaCl to simulate physiological conditions and establish charge neutrality. Energy minimization employed the steepest descent method until the system reached energy convergence.

The pre-equilibration phase consisted of consecutive 100 ps simulations using NVT and NPT ensembles, and with the temperature maintained at 310 K. We applied harmonic constraints of 1000 kJ mol^−1^ nm^−2^ to solute heavy atoms during this phase. Production simulations ran for 100 ns with all bond lengths constrained by the Linear Constraint Solver (LINCS) algorithm. Long-range electrostatic interactions utilized the particle-mesh Ewald (PME) method, while van der Waals interactions employed the Verlet Method. System temperature remained at 310 K through a velocity-rescaling thermostat, and pressure was controlled at 1 atm used a Parrinello-Rahman barostat.^54^^,^^55^^,^^56^

Trajectory analysis incorporated several measurements. Specifically, we calculated root-mean-square deviation for receptor Cα atoms and ligand heavy atoms relative to initial configurations using gmx rms. The radius of gyration, for each complex was determined through gmx gyrate, and intermolecular atomic contacts within 0.6 nm were quantified using gmx mindist. For binding free energy evaluation, we extracted 1000 equally spaced conformations from the equilibrated trajectory segment spanning 10–100 ns. Binding free energies were calculated with the MM - GBSA method in gmx_MMPBSA using default parameters.^57^

#### Computational Identification of Key HPCTP-Binding Residues in SLC2A1 and SLC6A3

Based on the per-residue energy decomposition analysis, the five residues of SLC2A1 that contributed most to HPCTP binding were individually mutated to alanine, yielding the T137A, P141A, H160A, Q161A, and W388A mutants. Similarly, the five most contributing residues of SLC6A3 were mutated to alanine to generate the R85A, L89A, T316A, H477A, and R544A mutants. For each mutant complex, 100 ns molecular dynamics simulations were performed, followed by binding free energy calculations, to evaluate the impact of these key residue mutations on the stability and binding affinity of SLC2A1 and SLC6A3 toward HPCTP.

#### Glucose uptake assay

The hCMEC/D3 cells were seeded into 12-well plates with 4×10^5^ cells per well and cultured in complete medium at 37°C, 5% CO_2_ incubator for 24 h before exposure. HPCTP exposure concentrations were set at 0, 1, 10, and 100 nM. The 0 nM group contained 0.1% DMSO as the vehicle control.

After 24 h of exposure, 2-NBDG dye was added according to the manufacturer’s instructions. 2-2-NBDG is a fluorescently labeled glucose analog that is taken up by cells via glucose transporters (GLUTs). Following co-incubation of 2-NBDG with the cells for 6 h, fluorescent images were captured using a Carl Zeiss AG fluorescence microscope. Integrated Density values were quantified using ImageJ software. The results were subsequently normalized to obtain the relative fluorescence intensity ratios between the treatment groups and the control group.

#### Glucose permeability assay

6.5 mm Transwell chambers with 0.4 μm pore polycarbonate membranes were placed into 24-well plates, and hCMEC/D3 cells were seeded using the same procedure described above at a density of 2 × 104 cells per well, culturing for 48 h until cells complete confluence was achieved. Cells were exposed to HPCTP at concentrations of 0, 1, 10, and 100 nM for 24h with 400 μL exposure medium in the upper and lower layers of each Transwell insert. After exposure, 400 μL of glucose-containing complete medium (60 mM) was added to the upper chamber, while an equal volume of glucose-free complete medium was added to the lower chamber. Following 6h of incubation, medium from both the upper and lower chambers as well as cell lysates were collected for analysis, respectively. Glucose concentrations in the samples were determined using an enzyme-linked immunosorbent assay (ELISA). The Optical Density (OD) values of all samples were measured at 450 nm. A standard linear regression curve was generated by plotting the concentrations of the standards against their corresponding OD values, and glucose concentrations in the samples were subsequently calculated based on this calibration curve. The obtained concentrations were normalized to generate relative percentage values for the treatment groups compared with the control group.

#### Reverse transcription quantitative polymerase chain reaction

Total RNA from hCMEC/D3 cells was extracted according to the instructions of the Servicebio Cell Total RNA Extraction Kit. Reverse transcription quantitative polymerase chain reaction (RT-qPCR) was performed according to the reagent kit instruction manual. Amplification was carried out using a Bio-Rad PCR system with an annealing temperature of 60 °C. The human-specific primers for SLC2A1 mRNA were as follows: forward, 5′-TGAGCATCGTGGCCATCTTT-3′; reverse, 5′-CCGGAAGCGATCTCATCGAA-3′. GAPDH was used as the endogenous control, with the following primers: forward, 5′-AATGGGCAGCCGTTAGGAAA-3′; reverse, 5′-GCGCCCAATACGACCAAATC-3′.

### Quantification and statistical analysis

Statistical analyses were performed using SPSS. Comparisons among multiple groups were performed using one-way analysis of variance (ANOVA). Significance levels were denoted by ∗*p* < 0.05, and standard error was presented as mean ± standard error of the mean (SEM).
